# Mixed Lineage Leukemia 5 (MLL5) Protein Stability Is Cooperatively Regulated by O-GlcNac Transferase (OGT) and Ubiquitin Specific Protease 7 (USP7)

**DOI:** 10.1371/journal.pone.0145023

**Published:** 2015-12-17

**Authors:** Xiaodan Ding, Wei Jiang, Peipei Zhou, Lulu Liu, Xiaoling Wan, Xiujie Yuan, Xizi Wang, Miao Chen, Jun Chen, Jing Yang, Chao Kong, Bin Li, Chao Peng, Catherine C. L. Wong, Fajian Hou, Yan Zhang

**Affiliations:** 1 Department of Immunology, Nanjing Medical University, Jiangsu, China; 2 Key Laboratory of Molecular Virology and Immunology, Institut Pasteur of Shanghai, Chinese Academy of Sciences, Shanghai, China; 3 Shanghai Red House Obstetrics and Gynecology Hospital, Fudan University, Shanghai, China; 4 Institute of Biology and Medical Sciences, Soochow University, Jiangsu, China; 5 College of life science, Sun Yet-Sen University, Guangzhou, China; 6 College of Life Science, Shanghai Normal University, Shanghai, China; 7 National Center for Protein Science Shanghai, Institute of Biochemistry and Cell Biology, Shanghai Institutes for Biological Sciences, Chinese Academy of Sciences, Shanghai, China; The University of Hong Kong, HONG KONG

## Abstract

Mixed lineage leukemia 5 (MLL5) protein is a trithorax family histone 3 lysine 4 (H3K4) methyltransferase that regulates diverse biological processes, including cell cycle progression, hematopoiesis and cancer. The mechanisms by which MLL5 protein stability is regulated have remained unclear to date. Here, we showed that MLL5 protein stability is cooperatively regulated by O-GlcNAc transferase (OGT) and ubiquitin-specific protease 7 (USP7). Depletion of OGT in cells led to a decrease in the MLL5 protein level through ubiquitin/proteasome-dependent proteolytic degradation, whereas ectopic expression of OGT protein suppressed MLL5 ubiquitylation. We further identified deubiquitinase USP7 as a novel MLL5-associated protein using mass spectrometry. USP7 stabilized the MLL5 protein through direct binding and deubiquitylation. Loss of USP7 induced degradation of MLL5 protein. Conversely, overexpression of USP7, but not a catalytically inactive USP7 mutant, led to decreased ubiquitylation and increased MLL5 stability. Co-immunoprecipitation and co-immunostaining assays revealed that MLL5, OGT and USP7 interact with each other to form a stable ternary complex that is predominantly located in the nucleus. In addition, upregulation of MLL5 expression was correlated with increased expression of OGT and USP7 in human primary cervical adenocarcinomas. Our results collectively reveal a novel molecular mechanism underlying regulation of MLL5 protein stability and provide new insights into the functional interplay among O-GlcNAc transferase, deubiquitinase and histone methyltransferase.

## Introduction

MLL5 protein, a trithorax group protein and histone 3 lysine 4 (H3K4) methyltransferase, was originally identified in a segment of chromosome band 7q22 that is frequently deleted in human myeloid leukemia [1,2]. Previous studies suggest that MLL5 is an important regulator of the cell cycle progression, either knockdown or overexpression of the MLL5 protein in cells causes aberrant cell cycle progression [3–5]. Several studies using *Mll5*-deficient mice have strongly suggested a crucial role in both hematopoiesis and spermatogenesis [6–9]. Moreover, MLL5 isoforms appear to be involved in human papillomavirus (HPV) viral E6 and E7 protein transcription in HPV16/18-associated cervical cancers and natural killer cell-mediated innate anti-tumor immunity [10,11]. Recent clinical research indicates that higher expression of MLL5 serves as a useful biomarker for predicting survival outcomes in patients with acute myeloid leukemia after chemotherapy treatment [12–14]. Interestingly, a recent whole-exome sequencing study linked MLL5 to autism spectrum disorder, a serious neurodevelopmental disorder [15]. Despite its extensive physiological and pathological functions, the mechanisms regulating MLL5 protein stability remain largely unknown.

The hexosamine biosynthetic pathway (HBP) is an important metabolic signaling mechanism that regulates post-translational modification of cytoplasmic and nuclear proteins by O-linked β-N-acetylglucosamine (O-GlcNAc) [16]. The level of uridine diphosphate N-acetylglucosamine (UDP-GlcNAc), a end product of the HBP pathway and the immediate donor substrate for O-GlcNAcylation, is modulated by the availability of glucose, fatty acids, amino acids, and nucleotides. Therefore, O-GlcNAcylation is considered a nutrient sensor that regulates nearly all aspects of cellular metabolism [17]. O-GlcNAc transferase (OGT), the sole enzyme responsible for O-GlcNAcylation in mammalian cells, utilizes UDP-GlcNAc to catalyze addition of the O-GlcNAc group into hydroxyl groups of serine or threonine residues of target proteins [18]. So far, more than thousands of cytoplasmic and nuclear proteins identified as O-GlcNAcylated by OGT [19] are implicated in many aspects of cellular processes, such as gene transcription and translation, nutrient sensing, neuronal function, cell cycle progression, lymphocyte activation, and stress response [20–31]. Consistently, deletion of OGT in mice has led to early embryonic lethality and defective somatic and reproductive cell development [32,33]. Importantly, abnormal O-GlcNAcylation contibutes to the etiology of several human diseases, such as diabetes, neurodegenerative disorders, heart disease and certain cancers [34–47]. O-GlcNAcase (OGA) catalyzes the reverse reaction to remove the O-GlcNAc modification from substrate proteins. Genetic disruption of OGA in mice has been shown to induce neonatal lethality with developmental delay and defects in metabolic homeostasis [48,49]. Therefore, OGT and OGA appear to regulate highly dynamic and reversible cycling of O-GlcNAcylation in response to metabolic changes in cells.

Ubiquitylation and proteasome-mediated degradation are important regulatory mechanisms for protein stability and function. Ubiquitylation levels of proteins are controlled *via* balance between E1, E2 and E3 ubiquitinating enzymes and deubiquitinating enzymes [50]. Ubiquitin-specific protease 7 (USP7) belongs to the ubiquitin-specific protease family of deubiquitinating enzyme and plays a complex role in regulating the stability of tumor suppressor p53 and its E3 ubiquitin ligase, MDM2 [51–53]. Later studies disclosed that USP7 is a critical regulator of the activities of proteins involved in DNA damage response, immune response, signal transduction, neuronal differentiation and epigenetic modulation [54–66].

In the current study, we showed that OGT and USP7 interact with MLL5 protein to form a stable protein complex in the cell nucleus. OGT and USP7 maintain the stability of MLL5 protein by inhibiting its ubiquitylation and degradation. Absence of either OGT or USP7 triggers rapid degradation of MLL5 proteins *via* the ubiquitin-proteasomal pathway. Notably, upregulation of MLL5 is correlated with increased expression of OGT and USP7 in human primary cervical adenocarcinomas. Our results collectively demonstrate a novel molecular mechanism of MLL5 protein stabilization, along with significant associations among cell metabolic sensors, protein deubiquitinase and histone methyltransferase.

## Materials and Methods

### Cell culture and transfection

HEK293T and HeLa cells (from ATCC) were cultured in DMEM (Gibco) supplemented with 10% FBS (Hyclone), non-essential amino acids (Gibco) and 2-mercaptoethanol (Pierce). HeLa cells were transfected with plasmids using Lipofectamine 2000 (Invitrogen) under the instruction of manufacturers. HEK293T cells were transfected using PEI (MW-25000, Polysciences).

### Co-Immunoprecipitation and western blotting

48h post transfection, HEK293T cells were washed with phosphate-buffered saline (PBS) and lysed in cell lysis buffer (1% NP-40, 20mM HEPES (pH7.5), 20mM KCl, 150mM NaCl, 5mM EDTA, 1mM Na_3_VO_4_ and complete protease inhibitor cocktails (04693132001, Roche)). Cell lysates were incubated on ice for 30min, then incubated with antibody for 14h at 4°C and protein A/G plus agarose (SC-2003, Santa Cruz) beads for another 1h at 4°C. The beads were washed 3 times with cell lysis buffer and boiled with loading buffer before western blotting analysis. For analysis of post-translational modifications of proteins, the cells were lysed using lysis buffer as below: 1% NP-40, 0.1% SDS, 20mM HEPES (pH7.5), 20mM KCl, 300mM NaCl, 5mM EDTA, 1mM Na_3_VO_4_ and complete protease inhibitor cocktails.

### Ubiquitin-His pull down assay

Cells for Ubiqutin-His pull down were treated with 5μM MG132 for 12h before harvesting. Cells were then lysed in pH8.0 urea lysis buffer(8M Urea, 100mM Na_2_HPO_4_,10mM Tris-HCl,pH = 8.0, 0.2%TritonX-100, 10mM Imidazole) for 20 min. Spin at 12000rpm, 30min. Cell lysates were incubated with Ni-NTA beads (30210, Qiagen) for 4h at room temperature. The beads were sequentially washed twice with pH 8.0 urea buffer, pH 6.3urea buffer (8M Urea, 100mM Na_2_HPO,10mM Tris-HCl,pH = 6.3, 0.2%TritonX-100, 10mM Imidazole) and pH 8.0 wash buffer(10mM Tris-HCl,pH = 8.0, 100mM NaCl,20% Glycerol,1mM DTT, 10mM Imidazole). At last, preloading buffer (62.5 mM Tris-HCl, pH6.8, 15%SDS, 8M Urea, 10% glycerol, 100mM DTT) and 5×loading buffer was added and incubated for 30min at 30°C. The samples were separated on SDS-PAGE gel.

### Plasmids, antibodies and reagents

pCDNA3 and pCDEF3 vectors obtained from Invitrogen and Addgene, respectively. Ubiqutin-His, Ubiqutin-HA, pIP-Usp7-Myc and pIP-Usp7 (C223S)-Myc plasmids were gifts from Dr. Bin Li. HA-PRK-Usp7 plasmid was a gift from Dr. Xingzhi Xu. pCDNA3-OGT-H508A plasmid was a gift from Dr. Jiemin Wong. The antibodies used are listed below: anti-MLL5 (AP14173a, Abgent), anti-FLAG M2 (F1804, Sigma), anti-HA antibody (3724, CST), anti-V5 (sc-271944, Santa Cruz), anti-Myc (16286-1-AP, Proteintech), anti-beta-Actin (A5316, Sigma), anti-ubiquitin (sc-8017, Santa Cruz), anti-OGT (Ab96718, Abcam), anti-O-Lined N-Acetylglucosamine (RL2) (ab2739, Abcam), anti-USP7 (3277, CST), anti-p27 antibody (610241, BD), anti-p16 antibody (sc-55600, Santa Cruz), anti-p21 antibody (2947, CST), anti-p14 (2407, CST), anti-Cyclin E (4126, CST). MG132 was purchased from Selleckchem (S2619). Cycloheximide was purchased from Sigma (C7698).

### Lentiviral transduction and infection

ShRNAs were cloned into pLKO.1 vectors, then co-transfected into HEK293T cells with packaging plamids (pSPAX2 and pMD2.G). The supernatants were collected at 48h post transfection for infection. The HeLa cells were incubated with supernatants containing virus particles and supplemented 8μg/ml of protamine (P4020, Sigma) for 12h, then the medium was replaced with fresh medium. Puromycin or G418 were added to screen the cells at 36h post transfection. The Sh-OGT, Sh-USP7 and Sh-Scramble sequence were shown as below: Sh-OGT-1275: 5’-CAGTTCGCTTGTATCGTAAAGCAT T-3’, Sh-OGT-1412: 5’-CCTACCTTTGCTGATGCCTACTCTA-3’, Sh-USP7-1324: 5’-TCCTAAGGACCCTGAAATTA-3’, Sh-USP7-2426: 5’-TTGTGGTTACGTTATCAAATACTC-3’, Sh-Scramble: 5’- TTCTCC GAACGTGTCACGTAC-3’).

### Cell Proliferation, cell cycle and apoptosis assay

Six days after infection, 2×10^4^ HeLa cells were seeded in triplicate on 12-well plate and counted on consecutive six days. Cell cycle was measured using FITC-BrdU Flow Kit (559619, BD Pharmingen). The assay was followed by the manuafacture’s protocol as previously described [5]. Apoptosis assay was measured using FITC-Annexin V Apoptosis detection Kit (556547, BD Pharmingen). The progression was followed by previously described [67]. The staining cells was analysed by Flow Cytometry (BD Fortessa). Data analysis was done using FlowJo 7.6 software.

### Immunofluorescence and confocal microscopy

HeLa cells were co-transfected with HA-PRK5-USP7 and pCDEF3-MLL5-FLAG and pCDNA3-OGT-V5 plasmids using lipofectamine 2000 (Invitrogen). 48h after transfection, cells were fixed for 1h with 2% PFA and permeabilized with 1% Triton-X-100. Cells were then blocked with blocking buffer (0.5% Triton X-100, 3% BSA, 10% NCS in PBS) for 1h at room temperature. Cells were stained with monoclonal anti-FLAG (F1804, Sigma), monoclonal anti-HA (11867423001, Roche) and polyclonal anti-OGT (Ab96718, Abcam) antibodies. Donkey-anti-mouse Alexa Fluor-555 (A21422, Invitrogen), Donkey-anti-rabbti Alexa Fluor-488 (A110081, Invitrogen) and Donkey-anti-Rat Alexa Fluor-633 (A21082, Invitrogen) were used as secondary antibodies. Cell nuclei were stained with DAPI. The slides were imaged with a laser confocal microscope (Leica SP5).

### Immunoprecitpiation of MLL5

HEK293T cells were transfected with the constructs of pCDEF3-MLL5-3×FLAG or pCDEF3. The transfected cells were lysed with lysis buffer (1% NP-40, 20mM HEPES (pH7.5), 20mM KCl, 150mM NaCl, 5mM EDTA, 1mM Na_3_VO_4_ and complete protease inhibitor cocktails). Cell lysates were processed by centrifuge and supernatant was incubated with M2 FLAG affinity gel (A2220, Sigma) at 4°C for 12h. Bounded protein was washed four times with lysis buffer and rinsed with TBS once at last. Elution of 3×FLAG fusion proteins was executed by competition with 3× FLAG Peptide (F4799, Sigma).

### Assays for interaction of USP7, OGT and MLL5

HEK293T cells were transfected with the constructs of pCDEF3-MLL5-3×FLAG only or together with Lv-OGT-V5 and pIP-USP7-Myc. 48h post transfection, cells were treated like Co-immunoprecipitaion. The cell lysates were incubated with Anti-FLAG M2 affinity gel (A220,Sigma) overnight at 4°C. The resin was washed for four times with lysis buffer. 100μl PBS and 3μl of 5μg/μl of 3×FLAG peptide were added into the resin. The samples were incubated with gentle shaking for 1h at 4°C. the resin was centrifuged at 5200×*g* and the supernatants were transferred into fresh tubes. Superdex-200 3.2/300 gel filtration column was equilibrated with cell lysis buffer(1% NP-40, 20mM HEPES (pH7.5), 20mM KCl, 150mM NaCl, 5mM EDTA, 1mMNa3VO4). FPLC (GE Healthcare) and a Superdex-200 were used for protein purification. The fractions were eluted on the 96-well plate. The elution were assayed with Western Blotting.

### In solution tryptic digestion and HPLC-MS/MS

The MLL5 proteins solution were tryptically digested following the procedure described previously [68]. Briefly, the protein precipitate was resolved by 8M Urea, and then sequentially treated with 5mM TCEP (Tris(2-carboxyethyl)phosphine hydrochloride) and 10mM IAA (iodoacetamide) to reduce the di-sulfide bond and alkylate the resulting thiol group. The mixture was digested for 16hr at 37°C by trypsin at an enzyme-to-substrate ratio of 1:50 (w/w). The tryptic digested peptides were desalted with C18 Zip-Tips and then loaded on an in-house packed capillary reverse-phase C18 column (15cm length, 100μM ID × 360μM OD, 5μM particle size, 100Å pore diameter) connected to a Thermo Easy-nLC1000 HPLC system. The samples were analyzed with a 180min-HPLC gradient from 0% to 100% of buffer B (buffer A: 0.1% formic acid in Water; buffer B: 0.1% formic acid in 20/80 water/acetonitrile) at 300 nL/min. The eluted peptides were ionized and directly introduced into a Q-Exactive mass spectrometer using a nano-spray source. Survey full-scan MS spectra (from m/z 300–1800) was acquired in the Orbitrap analyzer with resolution r = 70,000 at m/z 400.

### Analysis of tandem mass spectra

Protein identification and post-translational modification analysis were done with Integrated Proteomics Pipeline—IP2 (Integrated Proteomics Applications, Inc., http://www.integratedproteomics.com) using ProLuCID/Sequest [69], DTASelect2 [70]. Spectrum raw files were extracted into ms2 files from raw files using RawExtract, and the tandem mass spectra were searched against the Uniprot human protein database; plus sequences of known contaminants such as keratin and porcine trypsin concatenated to a decoy database in which the sequence for each entry in the original database was reversed using ProLuCID/Sequest. Carbamidomethylation (+57.02146) of cysteine was considered as a static modification and we require 2 peptides per protein and at least one trypitic terminus for each peptide identification. Search space included all fully- and half-tryptic peptide candidates with missed cleavage restrictions. The ProLuCID search results were assembled and filtered using the DTASelect program (version 2.0) with false discovery rate (FDR) of 0.01; under such filtering conditions, the estimated false discovery rate was below ~1% at the protein level in all analysis.

### Cervical cancer specimens and immunohistochemistry

In this study, the cervical cancer surgical specimens were collected from Shanghai Red House Obstetrics and Gynecology Hospital of Fudan University. This study was approved by the Ethics Committee of Shanghai Red House Obstetrics & Gynecology Hospital, and written informed consent was provided by all patients. The specimens were fixed in 4% neutral buffered formalin for 48h at room temperature. The paraffin-embedded specimens were cut in 5-μm-thick sections and placed on slides. After deparaffinization and antigen retrieval, the slides were blocked in 5% horse serum diluted in PBS for 30min. The diluted primary antibodies of MLL5 (1:100, Ab7533, Abcam), OGT (1:100, Ab96718, Abcam) and USP7 (1:100, Ab4080, Abcam) were applied. The slides were incubated for 60min at room temperature in a humidified box. After washing 4 times with Tris-buffered saline, the sections were incubated with the biotinylated secondary antibody (SC-2030, Santa Cruz). The sections were visualized with 3, 3’-diaminobenzidine (DAB). The secions were counterstained with haematoxylin.

## Results

### OGT interacts with and stabilizes MLL5 protein

**Fig 1:**
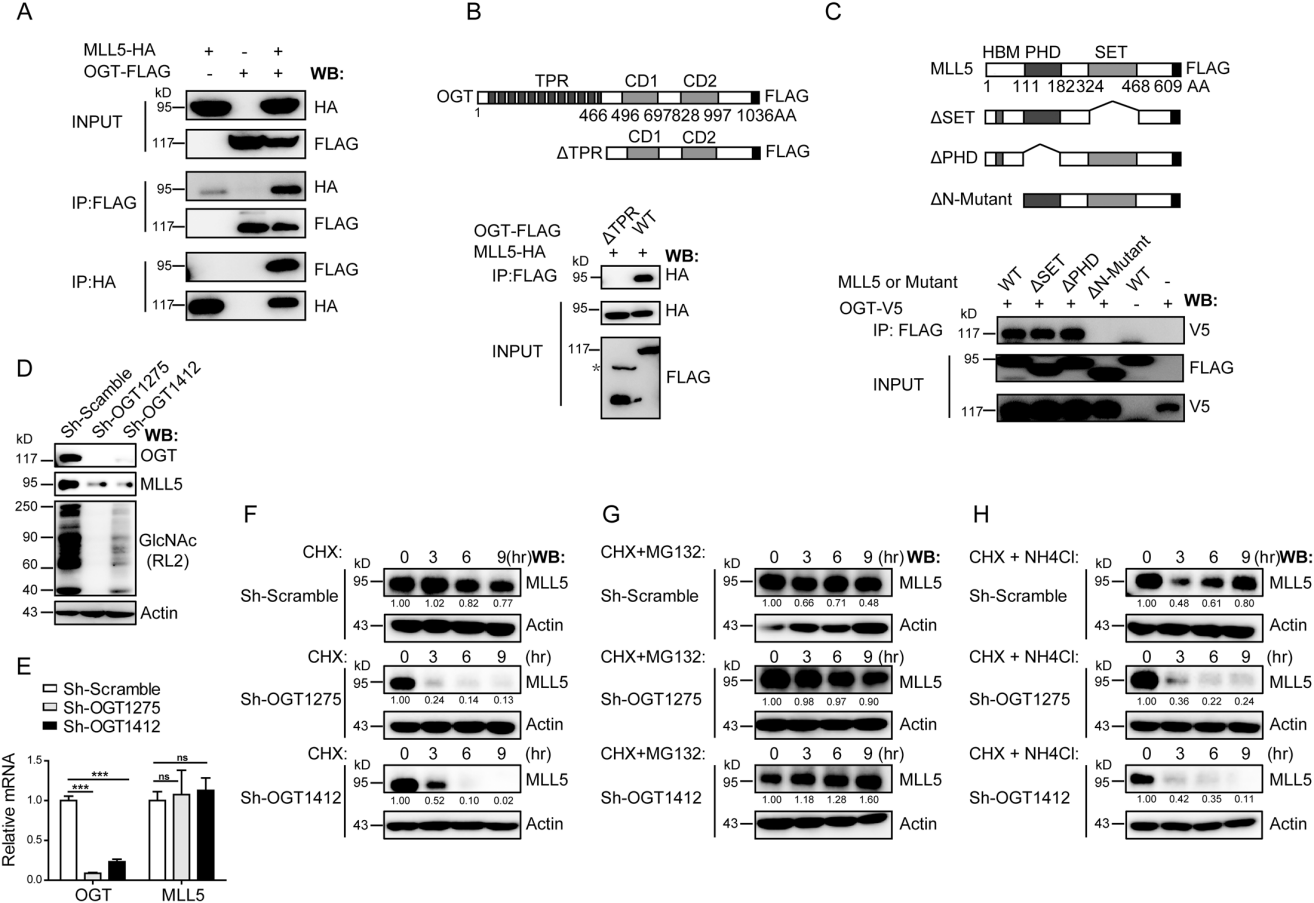
OGT interacts with and stabilizes MLL5 protein. **(A)** HEK293T cells were transiently co-transfected with expression vectors for HA-tagged MLL5 and FLAG-tagged OGT, and extracts prepared and immunoprecipitated with anti-HA or anti-FLAG antibodies. The presence of FLAG-tagged OGT or HA-tagged MLL5 protein were examined by western blotting using anti-FLAG or anti-HA antibodies, respectively. **(B)** Mapping the MLL5 interacting domains in the OGT protein. *Upper panel*, a schematic representation of the domain structure of the full length OGT and its truncated *Δ*TPR mutant. *Lower panel*, HEK293T cells were co-transfected with expression vectors for FLAG-tagged OGT or its truncated ΔTPR mutant and HA-tagged MLL5 and truncated mutants. 48h after transfection, cell lysates were harvested and FLAG-tagged OGT or its truncated *Δ*TPR mutant proteins were immunoprecipitated (IP) with anti-FLAG antibody, and the presence of MLL5 protein were examined by western blotting using anti-HA antibody. The asterisk indicate a nonspecific band. **(C)** Mapping the OGT interacting domains in the MLL5 protein. *Upper panel*, a schematic representation of the domain structure of the full length MLL5 and its truncated mutants. *Lower panel*, HEK293T cells were co-transfected with expression vector encoding MLL5-HA and OGT-FLAG or truncated mutants. Cells were lysed for co-immunoprecipitation as indicated. **(D)** Knockdown of OGT in HeLa cells leads to down-regulation of MLL5 protein. Two different lentivirus-based shRNAs target to an mRNA sequence located 1275 and 1412 bp downstream of the translation start site were used to knockdown OGT expression in HeLa cells. Whole cell lysates were harvested at day 6 post-infection. The expression of endogenous MLL5 and the global levels of O-GlcNAcylation were detected by anti-MLL5 and anti-GlcNAc antibodies. Actin was used as a loading control. **(E)** The mRNA levels of MLL5 remain unchanged by OGT knockdown. The mRNA levels of OGT and MLL5 in OGT knockdown cells were analyzed by quantitative RT-PCR. Two-tailed unpaired Student’s *t* tests were performed, p < 0.001. **(F-H)** Control or OGT knockdown cells were treated with CHX (100μg/ml), MG132 (30μM) or NH_4_Cl (5mM) for the indicated times before harvesting. The expression of endogenous MLL5 proteins were detected by anti-MLL5 antibody. β-actin was used as a loading control. Quantification of relative MLL5 levels is shown in the bottom panel. Numbers below lanes indicate densitometry of the protein presented relative to β-actin.

As a critical trithorax group histone methyltransnferase, MLL5 regulates a range of biological processes. However, the mechanisms regulating MLL5 protein stability remains to be clarified. OGT has been identified as a putative MLL5 binding partner[5]. To determine the biological significance of MLL5-OGT interactions, associations was initially confirmed using the co-immunoprecipitation assay (Fig 1A). Co-immunoprecipitation experiments using different OGT and MLL5 truncation mutants revealed that the TPR domains of OGT protein are required for binding to MLL5 (Fig 1B), whereas the N-terminal region (1–111 amino acids) of MLL5 paricipates in interactions with OGT (Fig 1C).

Next, we used a lentiviral-based short hairpin RNA (shRNA) approach to deplete OGT in HeLa cells and examined effects on MLL5 protein. As shown in Fig 1D and 1E, two different shRNAs targeted to mRNA sequences located 1275 and 1412 bp downstream of the translation start site resulted in effective elimination of OGT. Consistently, global levels of O-GlcNAcylation were markedly decreased in OGT knockdown cells (Fig 1D). Knockdown of OGT led to inhibition of cell proliferation and induction of apoptosis (S1A, S1C and S1D Fig), consistent with previous findings [47,71]. Moreover, expression levels of Cyclin E and several CDKIs, including p27, p21, p16 and p19 were increased in OGT knockdown cells (S1B Fig).

Unexpectedly, MLL5 protein levels were dramatically decreased in OGT knockdown cells (Fig 1D), although mRNA levels remained unchanged (Fig 1E). Accordingly, we speculated that regulation of MLL5 protein occurs at the post-translational, but not the transcriptional level. To further investigate whether OGT regulates the stability of MLL5, control or OGT knockdown cells were treated with cycloheximide (CHX), an inhibitor of protein biosynthesis, for the indicated times. As shown in Fig 1F, MLL5 protein levels were only slightly decreased after 9h of CHX treatment in control cells. In contrast, a significant decrease in the MLL5 protein level was observed in OGT knockdown cells treated with CHX, indicating that OGT regulates MLL5 stability by inhibiting its degradation. To further determine whether MLL5 protein is degaraded *via* the ubiquitin/proteasomal-mediated or the autophagy/lysosomal protein degradation pathway, control or OGT knockdown cells were treated with CHX plus the proteasome inhibitor, MG132, or the lysosome inhibitor, NH_4_Cl, for the indicated times. As shown in Fig 1G and 1H, MG132, but not NH_4_Cl treatment restored the protein levels of MLL5 after CHX treatment in OGT knockdown cells, suggesting that OGT maintains the stability of MLL5 protein mainly through inhibiting proteasomal-mediated degradation.

### OGT enhances MLL5 stability by inhibiting its ubiquitylation

**Fig 2:**
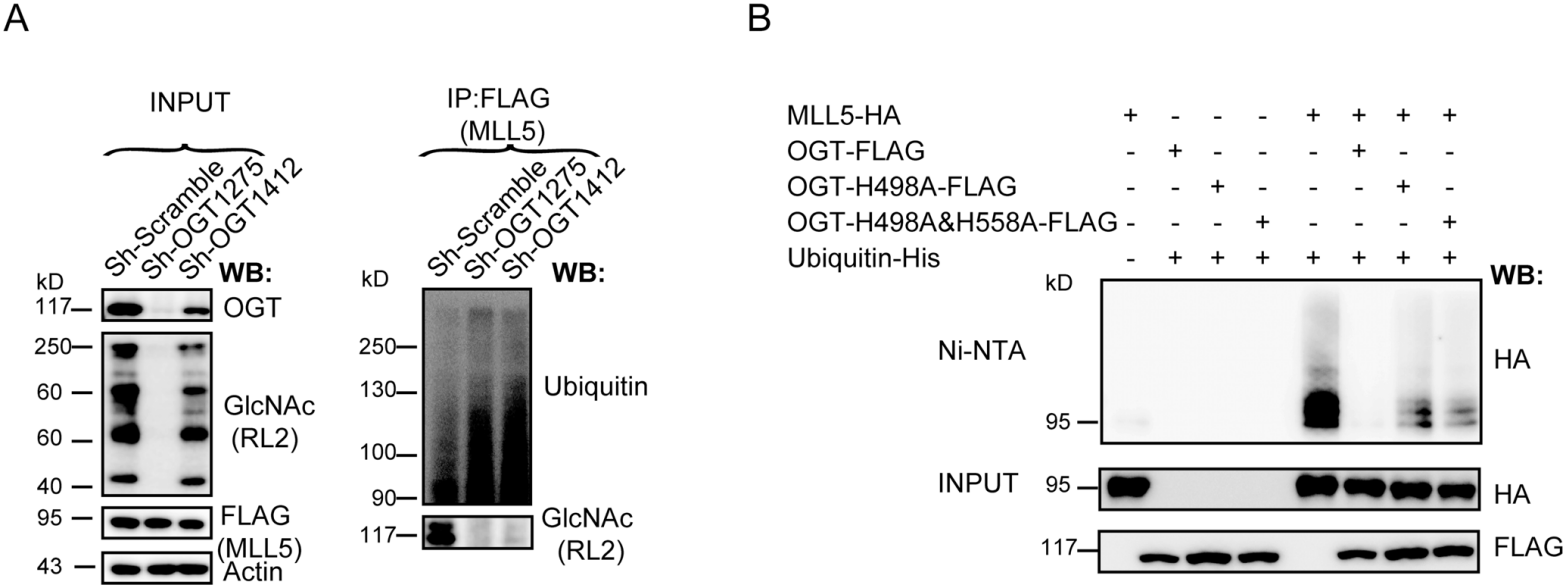
OGT enhances MLL5 stability by inhibiting its ubiqutiylation. **(A)** Knockdown of OGT in HeLa cells stably expressing FLAG-tagged MLL5. Cells were treated with MG132 (5μM) for 12h before harvest. FLAG-tagged MLL5 protiens were immunoprecipitated (IP) with anti-FLAG antibody, the levels of ubiqutiylation and O-GlcNAcylation were examined by western blotting using anti-ubiquitin or anti-GlcNAc antibodies, respectively. **(B) H**EK293T cells were co-transfected with plasmids encoding HA-tagged MLL5, His-tagged ubiquitin, and FLAG-tagged OGT or its enzymatic inactivating mutant OGT-H498A or OGT- H498A&H558A. The cells were treated with 5μM MG132 for 12h before harvesting. Ubiquitylated proteins were pulled down with Ni-NTA beads and the MLL5 was visualized by immunoblotting with by anti-HA antibody.

In view of the finding that MG132 treatment restores MLL5 protein levels in OGT knockdown cells, we further examined whether MLL5 protein is directly modified by ubiquitin and the effects of OGT knockdown on ubiquitylation levels of MLL5. To this end, HeLa cells stably over-expressing 3×FLAG-tagged MLL5 were infected with lentiviruses expressing two different OGT Sh-RNAs or a scrambled control ShRNA. After treatment with MG132, cell lysates were prepared and immunoprecipitated with anti-FLAG antibody, followed by SDS-PAGE separation and western blot analysis with anti-ubiquitin antibody. As shown in Fig 2A, knockdown of OGT resulted in an increase in the ubiquitylation of MLL5 protein.

To further investigate the potential role of OGT in MLL5 protein ubiquitylation, HEK293T cells were transiently co-transfected with expression vectors for HA-tagged MLL5, FLAG-tagged OGT and His-tagged ubiquitin and His pulldown experiments performed with Ni-NTA beads, followed by western blot analysis with anti-HA antibody. Overexpression of OGT protein induced a marked reduction in the ubiquitylation levels of MLL5 protein (Fig 2B), consistent with results from OGT knockdown cells.

Since OGT catalyzes addition of the O-GlcNAc group to the hydroxyl groups of serine or threonine residues of target proteins, we examined whether the O-GlcNAc transferase activity of OGT is required for inhibiting MLL5 ubiquitylation. To this end, expression vectors for FLAG-tagged OGT H498A or H498A and H558A double mutants [72] lacking catalytic activity and HA-tagged MLL5 were transiently co-transfected with that for His-tagged ubiquitin into HEK293T cells, and His pulldown experiment performed with Ni-NTA beads, followed by western blot analysis with anti-HA antibody. As shown in Fig 2B, in contrast to the wild-type OGT, overexpression of both OGT mutants caused only a slight decrease in the ubiquitylation of MLL5 protein, indicating that the O-GlcNAc transferase activity of OGT is required for preventing ubiquitylation of MLL5. In view of these finding, we propose that OGT enhances MLL5 protein stability by inhibiting its ubiquitylation and degradation.

### Indentification and charaterization of post-translational modifications of MLL5 protein with mass spectrometry

**Fig 3:**
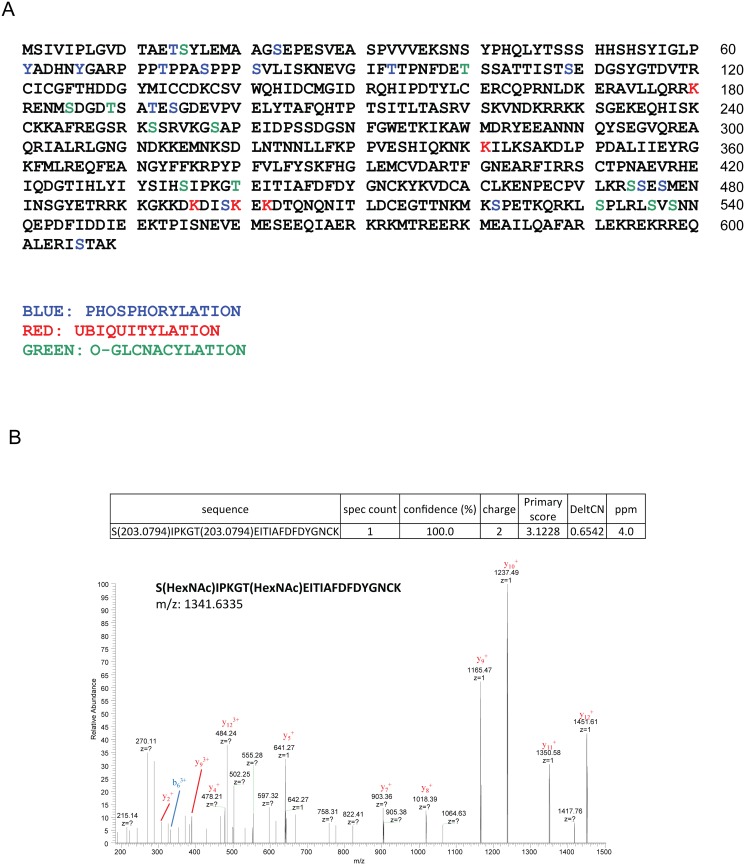
Characterization of post-translational modifications of MLL5 protein. **(A)** The phosphorylation sites were highlighted in blue, ubiquitylation sites in red, O-GlcNAcylation sites in green. **(B)** A representiative MS data of the N-GlcNAcylation at Ser435 and Thr440 were shown.

To further identify the putative post-translational modificatioins of MLL5, we systematically characterized potential post-translational modifications of the protein *via* mass spectrometry. An expression vector encoding 3×FLAG-tagged MLL5 protein was transiently transfected into HEK293T cells and co-immunoprecipitations performed with anti-FLAG antibody, followed by mass spectrum analysis. As shown in Fig 3A, multiple post-translational modifications, including O-GlcNAcylation, ubiquitylation and phosphorylation, were identified at various sites. A representiative mass spectrum data of the N-GlcNAcylation at Ser435 and Thr440 was shown in Fig 3B. Whether these post-translational modifications regulate MLL5 stability and function remains to be established.

### USP7 interacts with MLL5 protein

**Fig 4:**
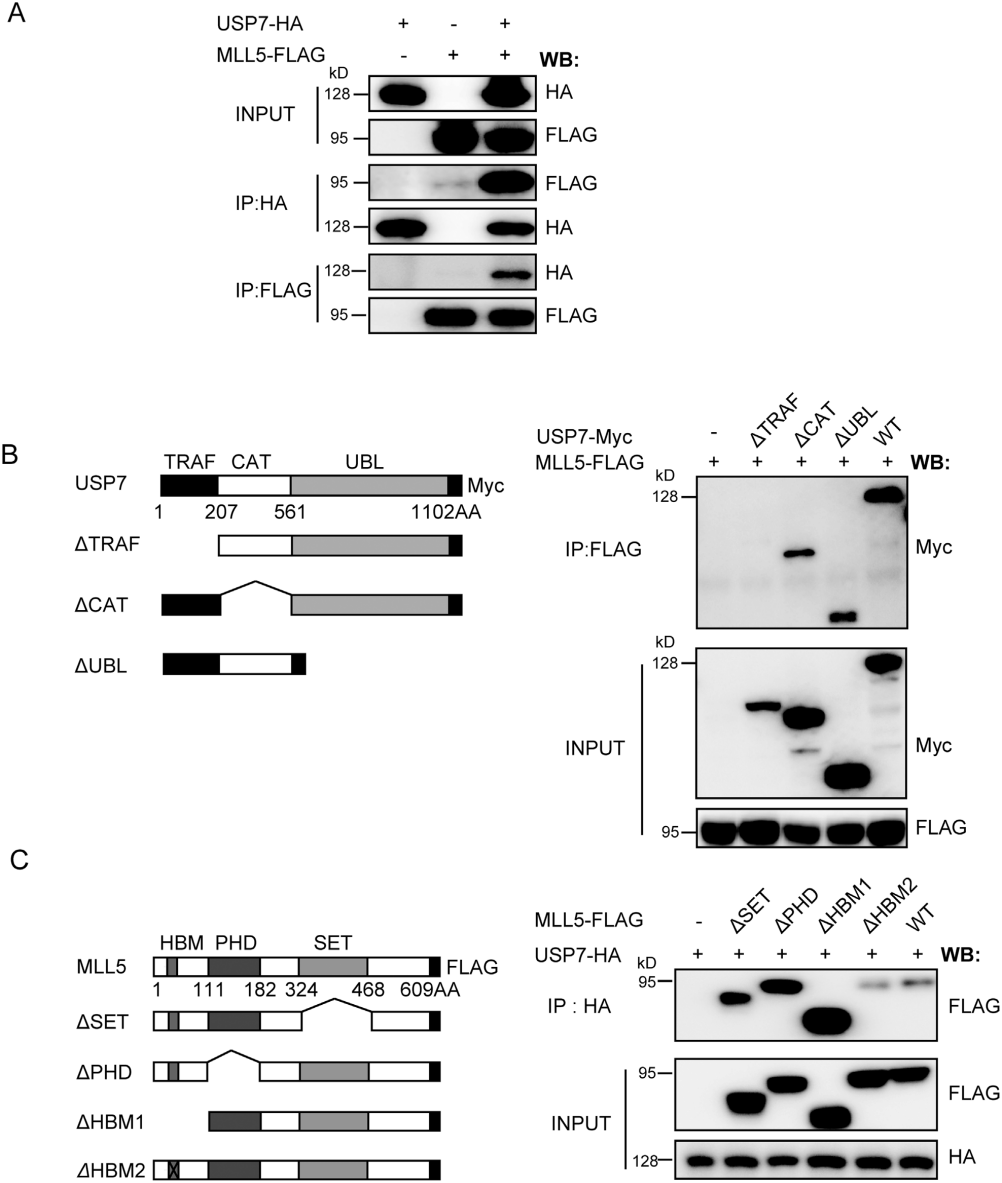
USP7 interacts with MLL5 protein. **(A)** The reciprocal immunoprecipitation of USP7 and MLL5. HEK293T cells were transiently co-transfected with plasmids encoding HA-tagged USP7 and FLAG-tagged MLL5. 48h after transfection, cells lysates were harvested and FLAG-tagged MLL5 or HA-tagged USP7 proteins were immunoprecipitated (IP) with anti-FLAG or anti-HA antibodies, and the presence of USP7 or MLL5 protein were examined by western blotting using anti-HA or anti-FLAG antibodies, respectively. **(B)** Mapping the MLL5 interacting domain in USP7 protein. *Left panel*, a schematic representation of the domain structure of the full length USP7 and its truncated mutants. *Right panel*, HEK293T cells were co-transfected with expression vectors encoding FLAG-tagged MLL5 and Myc-tagged USP7 or truncated mutants. FLAG-tagged MLL5 proteins were co-immunoprecipitated with anti-FLAG antibody, and the presence of USP7 protein and truncated mutants were examined by western blotting using anti-Myc antibody. **(C)** Mapping the USP7 interacting domain in MLL5 protein. *Left panel*, a schematic representation of the domain structure of the full length MLL5 and its truncated mutants. *Right panel*, HEK293T cells were co-transfected with expression vectors encoding HA-tagged USP7 and FLAG-tagged MLL5 or truncated mutants. HA-tagged USP7 or truncated mutants were co-immunoprecipitated with anti-HA antibody, and the presence of MLL5 protein was examined by western blotting using anti-FLAG antibody.

While our results showed that OGT increases MLL5 protein stability by inhibiting ubiquitylation and degradation, OGT itself lacks deubiquitinase activity. We speculated that factors other than OGT are involved in regulation of MLL5 ubiquitylation. To this end, MLL5 interacting protein were detected using the mass spectrometry. Expression vectors encoding 3×FLAG-tagged MLL5 protein were transiently transfected into HEK293T cells, and extracts prepared and immunoprecipitated with anti-FLAG antibody, followed by competitive elution using FLAG peptide and analysis by mass spectrometry analysis. Consequently, a number of putative MLL5-interacting proteins, including two deubiquitinases, USP7 and USP10 protein, were detected (S1 Table). Application of the co-immunoprecipitation assay to examine the interactions between the two deubiquitinases and MLL5 revealed that only USP7 interacts with MLL5 (Fig 4A and data not shown), which was therefore subjected to further analysis.

USP7, also designated HAUSP, contains N-terminal TRAF, intermediate CAT, and C-terminal UBL domains. The USP7 domain(s) responsible for interactions with MLL5 were investigated by using the co-immunoprecipitation assay. Truncated USP7 mutant lacking the TRAF domain abolished the capacity for MLL5 binding (Fig 4B), suggesting that this region mediates interactions with MLL5 protein. The co-immunoprecipitation assay was additionally performed to map USP7-interacting domains within the MLL5 protein. All MLL5 truncation constructs were able to interact with USP7 protein, as shown in Fig 4C, indicating that multiple domains or motifs of MLL5 protein may contribute to USP7 binding.

### USP7 deubiqutinates MLL5 protein

**Fig 5:**
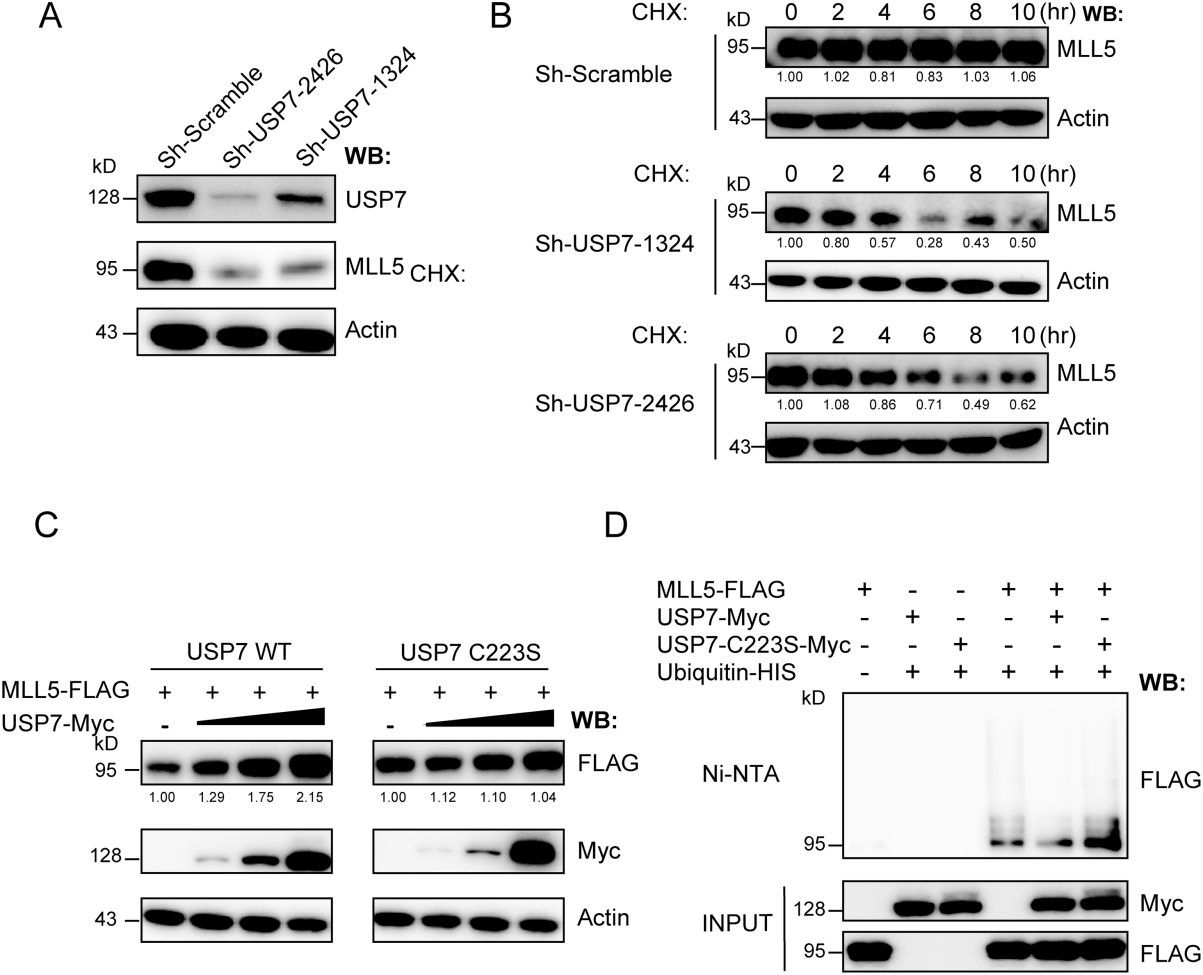
USP7 deubiquitinates MLL5 protein. **(A)** Knockdown of USP7 in HeLa cells leads to down-regulation of MLL5 protein. Two different lentivirus-based shRNAs target to an mRNA sequence starting 1324 and 2426 bp downstream of the translation start site were used to knockdown USP7 expression in HeLa cells. Whole cell lysates were harvested at day 6 post-infection. The expression of endogenous USP7 and MLL5 proteins were detected by anti-USP7 and anti-MLL5 antibodies. Actin was used as a loading control. **(B)** Control or OGT knockdown HeLa cells were treated with CHX (100μg/ml) for the indicated times before harvesting. The expression of endogenous MLL5 proteins were detected by anti-MLL5 antibody. Actin was used as a loading control. **(C)** HEK293T cells were co-transfected with plasmids encoding FLAG-tagged MLL5 (2μg) along with increasing amount of Myc-tagged USP7 or its inactive C223S mutant (0, 0.1, 0.5, 2μg). The presence of MLL5 and USP7 or inactive C223S mutant proteins were examined by western blotting using anti-FLAG or anti-Myc antibodies. **(D)** HEK293T cells were transiently co-transfected with plasmids encoding His-tagged ubiquitin, FLAG-tagged MLL5, Myc-tagged USP7 or its inactive C223S mutant. The cells were treated with MG132 (5μM) for 12h before harvesting, and the ubiquitylated proteins were pulled down with Ni-NTA beads and the MLL5 was visualized by immunoblotting with by anti-FLAG antibody.

To determine whether USP7 deubiquitinates MLL5 protein, we used a lentiviral-based shRNA approach to eliminate USP7 expression in HeLa cells, and examined the effects on MLL5 protein. Two different shRNAs targeted to mRNA sequences located 1324 and 2426 bp downstream of the translation start site were used, which effectively led to USP7 protein knockdown (Fig 5A). Notably, endogenous MLL5 protein levels were significantly decreased upon USP7 knockdown. Moreover, control or USP7 knockdown cells were treated with cycloheximide (CHX), and endogenous MLL5 protein levels evaluated for the indicated times. As shown in Fig 5B, a significant increase in the rate of MLL5 turnover was observed in USP7 knockdown cells following CHX treatment.

We additionally evaluated the impact of USP7 overexpression on MLL5 protein stability. HEK293T cells were transiently co-transfected with 3×FLAG-tagged MLL5 plasmid and various amounts of Myc-tagged USP7 plasmid, and levels of ectopic MLL5 protein were detected *via* western blot using anti-FLAG antibody. Protein levels of ectopic MLL5 were significantly increased with USP7 expression (Fig 5C). In contrast, the ectopic MLL5 protein level remained unchanged when catalytically inactive C223S mutant USP7 was used. The ubiquitylation levels of MLL5 protein in the presence wild-type or C223S mutant USP7 were further examined. Briefly, HEK293T cells were transiently co-transfected with expression vectors for 3×FLAG-tagged MLL5, Myc-tagged wild-type or C223S mutant USP7 and His-tagged ubiquitin. Co-immunoprecipitation experiments were performed using Ni-NTA beads and levels of MLL5 ubiquitylation evaluated *via* western blot with anti-FLAG antibody. As shown in Fig 5D, MLL5 ubiquitylation was dramatically decreased by wild-type USP7, but not the C223S mutant, indicating that stabilization of MLL5 by USP7 requires deubiquitylating enzymatic activity. Our data suggest that USP7 is crucially involved in both deubiquitylation and the stabilization of MLL5 protein.

### MLL5 forms a ternary complex with OGT and USP7

**Fig 6:**
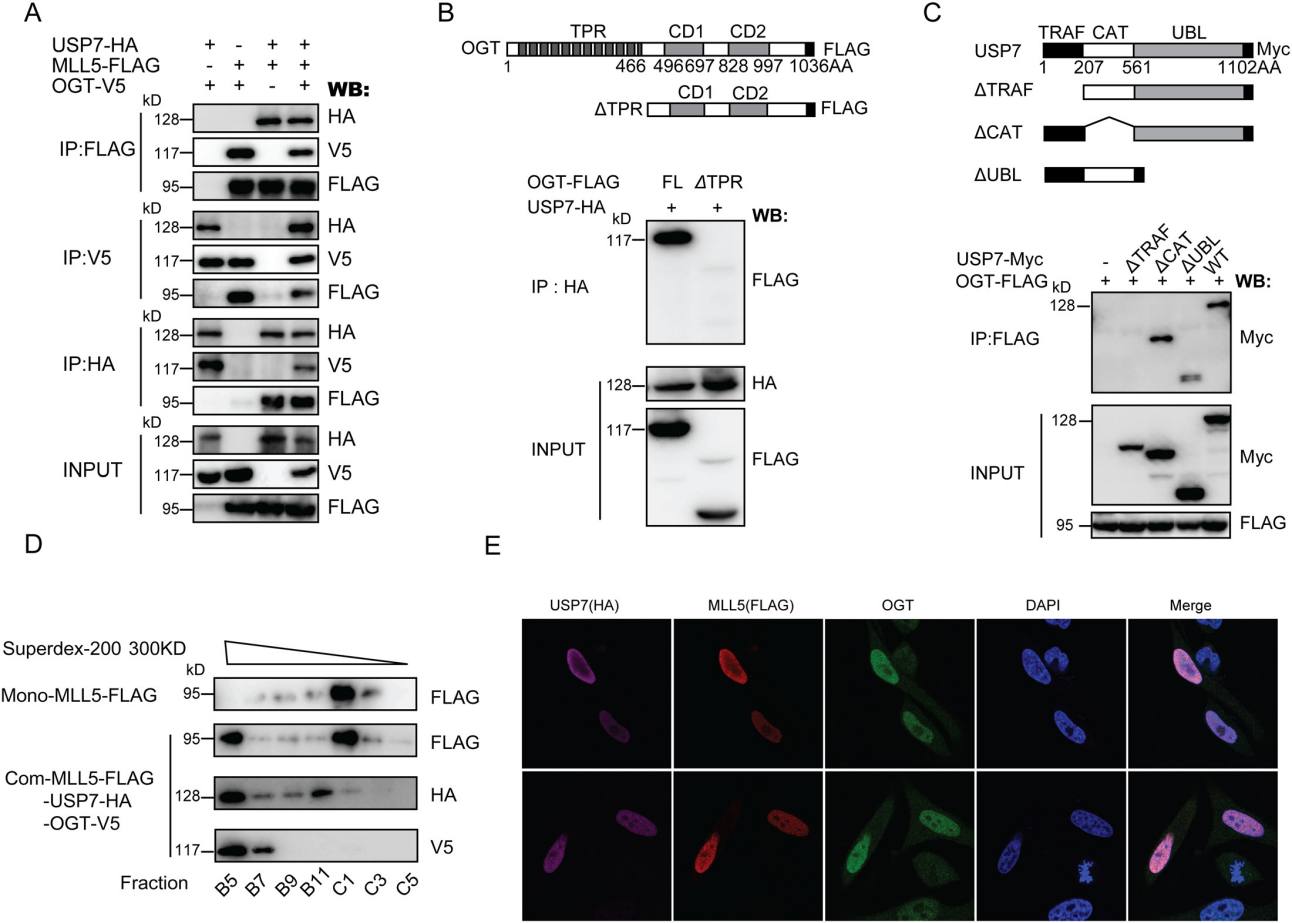
MLL5 forms a complex with OGT and USP7. **(A)** The reciprocal immunoprecipitation of MLL5, OGT and USP7. HEK293T cells were transiently co-transfected with plasmids encoding FLAG-tagged MLL5, V5-tagged OGT and HA-tagged USP7. 48h after transfection, cell lysates were harvested, and co-immunoprecipitation assays were performed as indicated. **(B)** Mapping the USP7 interacting domain in the OGT protein. *Upper panel*, a schematic representation of the domain structure of the full length OGT and its *Δ*TPR truncated mutant. *Lower panel*, HEK293T cells were transiently co-transfected with expression vectors for HA-tagged USP7 and FLAG-tagged OGT or its truncated *Δ*TPR mutant. 48h after transfection, the cells lysates were harvested and HA-tagged USP7 proteins were immunoprecipitated (IP) with anti-HA antibody, and the presence of OGT or its truncated *Δ*TPR mutant were examined by western blotting using anti-FLAG antibody. **(C)** Mapping the OGT interacting domain in the USP7 protein. *Upper panel*, a schematic representation of the domain structure of the full length USP7 and its truncated mutants. *Lower panel*, HEK293T cells were transiently co-transfected with expression vectors for FLAG-tagged OGT and Myc-tagged USP7 or its truncated mutants. 48h after transfection, cell lysates were harvested and FLAG-tagged OGT proteins were immunoprecipitated (IP) with anti-FLAG antibody, and the presence of USP7 or its truncated mutants were examined by western blotting using anti-Myc antibody. **(D)** Western blot profiles of selected fractions are shown. The bulk of MLL5 protein was found in the fraction C1, MLL5 protein were also presented in fraction B5, where OGT and USP72 were also present. **(E)** HeLa cells were transiently co-transfected with plasmids encoding FLAG-tagged MLL5, V5-tagged OGT and HA-tagged USP7, and then fixed and subjected to immunofluorescence using anti-FLAG monoclonal antibody to detect MLL5, anti-OGT polyclonal antibody to detect OGT, and anti-HA monoclonal antibody to detect USP7. Cells were stained with DAPI to highlight the nuclei.

Since both OGT and USP7 interact with and stabilize MLL5 protein, we examined whether these proteins form a complex. To this end, expression vectors for 3×FLAG-tagged MLL5, HA-tagged USP7 and V5-tagged OGT protein were transiently co-transfected into HEK293T cells, and the extracts prepared and subjected to immunoprecipitation with anti-FLAG, anti-HA or anti-V5 antibody, followed by western blot with the appropriate antibodies. Data from Fig 6A show that MLL5, OGT and USP7 proteins specifically co-immunoprecipitate with each other and form a ternary complex. Further analysis of the domains or motifs responsible for interactions between OGT and USP7 revealed that the TPR domain of OGT and TRAF domain of USP7 mediate the protein-protein interactions (Fig 6B and 6C).

Next, size exclusion chromatography was used to confirm the formation of the ternary complex of MLL5, OGT, and USP7. Expression vectors for 3×FLAG-tagged MLL5, HA-tagged USP7 and V5-tagged OGT protein were transiently co-transfected into HEK293T cells, and the extracts prepared and subjected to immunoprecipitation with anti-FLAG affinity gel. The samples was loaded onto a previously calibrated Superdex-200 3.2/300 gel filtration column. The fractions were eluted on the 96-well plate, and the elution were assayed by Western Blot with the appropriate antibodies. As shown in Fig 6D, although the bulk of MLL5 protein was found in the fraction C1, MLL5 protein were also presented in fraction B5, where OGT and USP72 were also present. This observation suggested that the three proteins formed a ternary complex.

Finally, we analyzed the subcellular localization patterns of the three proteins to ascertain whether they colocalize within cells. HeLa cells were transiently co-transfected with expression plasmids encoding the three proteins, and MLL5, OGT and USP7 localization detected *via* immunofluorescence staining using mouse anti-FLAG (M2) and rabbit anti-OGT polyclonal, and rat anti-HA antibodies, respectively. As shown in Fig 6E, all three proteins were predominantly detected within the cell nucleus. The results collectively suggest that MLL5 forms a ternary complex with OGT and USP7 and maintains its stability and function by preventing ubiquitylation and degradation.

### MLL5 protein levels are correlated with expression of OGT and USP7 in primary cervical adenocarcinomas

**Fig 7:**
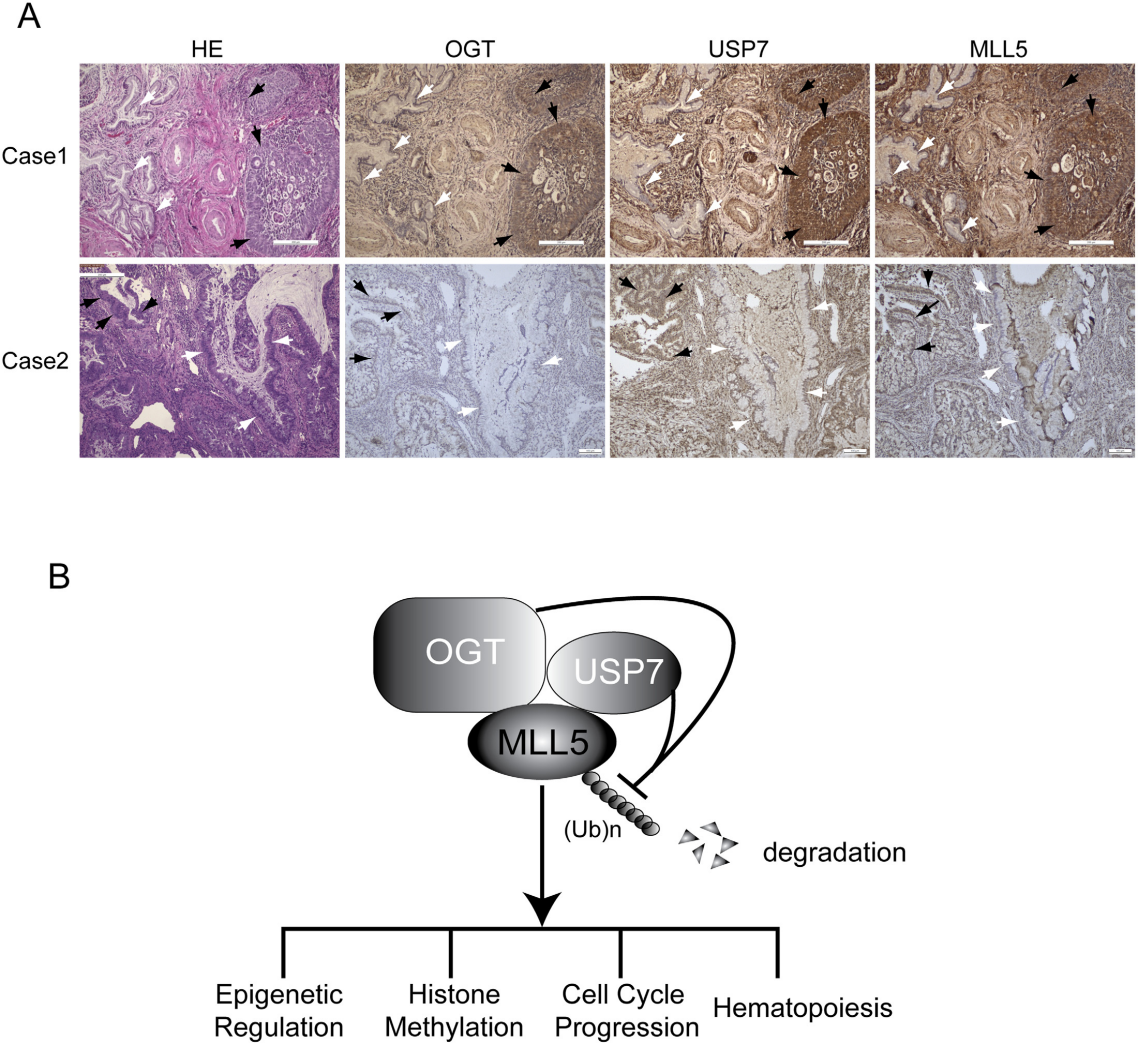
MLL5 protein levels in primary cervical adenocarcinomas correlated with expression of OGT and USP7. **(A)** Representative images of haematoxylin and eosin along with immuno-histochemical staining with antibodies against OGT, USP7 and MLL5 on paraffin-embedded sections of human primary cervical adenocarcinomas. Scale bars, 200 μm. Adenocarcinomas tissues were indicated with black arrows, and adjacent normal adenoid tissues were indicated with white arrows. **(B)** Model for OGT and USP7 cooperatively regulate the stability of MLL5 protein by inhibiting its ubiquitylation and degradation.

Increased levels of OGT or USP7 are associated with human cancers [73–76]. Since MLL5 stability is regulated by OGT and USP7, we determined whether MLL5 protein levels are also increased in human primary tumors. Two main cervical cancer sub-types have been identified at the clinic, specifically, cervical squamous cell carcinoma and adenocarcinoma. Levels of MLL5, OGT and USP7 in 8 pairs of human cervical squamous cell carcinoma and 2 pairs of human cervical adenocarcinoma and adjacent normal tissues were analyzed *via* immunostaining with the appropriate antibodies. Increased OGT and USP7 protein levels were primarily detected in cervical adenocarcinoma, but not cervical squamous cell carcinoma tissues (Fig 7A and data not shown). Interestingly, MLL5 protein levels were similarly increased in cervical adenocarcinomas, compared to adjacent normal tissues (Fig 7A). Our results suggest that increased levels of MLL5 protein are correlated with upregulation of OGT and USP7 in primary cervical adenocarcinomas.

## Discussion

Studies over recent years have greatly expanded our knowledge of epigenetic regulations mediated by MLL5 protein in gene transcription, cell cycle progression, hematopoiesis and tumorigenesis. Here, we showed that OGT stabilizes MLL5 protein by inhibiting its ubiquitylation. Moreover, USP7 was identified as a protein deubiquitinase that interacts with and deubiquitinases MLL5 protein. MLL5, OGT and USP7 formed a stable protein complex and colocalized in the cell nucleus. MLL5 protein levels were additionally correlated with increased OGT and USP7 expression in primary cervical adenocarcinomas. Data from the current study have thus revealed previously unknown mechanisms in maintenance of MLL5 protein stability involving regulation by OGT and USP7 (Fig 7B).

The majority of cell proteins undergo post-translational modifications (PTM) to extend their range of biological functions. Using the mass spectrometry, O-GlcNAcylation and ubiquitylation at different sites on MLL5 protein were identified. Our experiments further comfirmed that OGT plays a critical role in the maintenance of MLL5 protein stability. In the absence of OGT, MLL5 underwent rapid degradation through the ubiquitin proteasome pathway, while overexpression of wild-type but not mutant OGT lacking GlcNAcyltransferase activity induced a marked reduction in levels of MLL5 ubiquitylation, suggesting that the O-GlcNAcyltransferase activity of OGT is important in preventing ubiquitylation and degradation of MLL5. In addition to O-GlcNAcylation and ubiquitylation, phosphorylation at different sites on MLL5 protein were identified with our mass spectrum assay. Further determination of the effects of these PTMs on the stability and function of MLL5 protein would be of considerable interest.

In addition to the critical role of OGT, we showed that USP7, a protein deubiquitinase, is directly responsible for deubiquitination of MLL5 protein. USP7 was originally identified as a deubiquitinase for both tumor suppressor protein, p53, and its major E3 ligase, MDM2 [51–53]. Recent studies have implicated USP7 in a range of cellular processes, including DNA repair, signaling transduction, immune regulation and cell metabolism. In the current investigation, we showed that USP7 forms a stable complex with OGT and MLL5, with nuclear colocalization of all three proteins within the cell nucleus. Both USP7 and OGT proteins contain multiple motifs or domains with different functions. For example, the C-terminal UBL domain of USP7 protein is important for catalytic activity, whereas the N-terminal TRAF domain is critical for recruitment of target proteins [77]. Similarly, the N-terminal TPR domain of OGT is involved in recognition and binding of substrate proteins while the central catalytic domains (CD) of OGT are critical for the catalytic activity of the enzyme [78]. Using various truncation mutants and co-immunoprecipitation assays, we showed that the USP7 protein is associated with MLL5 and OGT mainly through the N-terminal TRAF domain, while OGT associates with USP7 and MLL5 through its N-terminal TPR domains. In contrast, multiple domains of MLL5 protein are required for association with OGT and USP7. Thus, OGT, USP7 and MLL5 form a highly organized protein complex through specific interactions among domains, and the absence of either USP7 or OGT leads to enhanced ubiquitylation and accelerated degradation of MLL5 protein. Our results raise the interesting question of whether OGT and USP7 catalyze and modulate each other’s activities. Further studies are required to clarify the interplay between these enzymes.

Upregulation of OGT and USP7 has been reported in a number of tumor types. These proteins potentially contribute to tumorigenesis either by modulating cellular metabolism or stability of the tumor suppressor, p53. Here, we detected increased protein expression of OGT, USP7 and MLL5 in human primay cervical adenocarcinomas, compared with adjacent normal tissues. Our results provide further evidence that OGT and USP7 cooperatively regulate stabilization of MLL5 protein. MLL5 protein is implicated in regulating cell cycle progression *via* the RB-E2F1 protein pathway [4,5]. Interestingly, recent studies have suggested that a MLL5 isoform is crucial for HPV E6 and E7 transcription and cervical cancer growth [11]. Therefore, increased MLL5 protein *via* upregulation of OGT and USP7 in tumor cells may contribute to altered cell proliferation and malignant transformation. The precise role of MLL5 protein in tumorigenesis requires further investigations.

In summary, OGT and USP7 cooperatively regulate the stability of MLL5 protein, a trithorax group H3K4 methyltransferase, through inhibiting its ubiquitylation and degradation. While the specific E3 ligase required for MLL5 ubiquitylation remains to be identified, our results indicate that USP7 acts as a *bona fide* MLL5 deubiquitinase. Considering that OGT is crucially involved in transcriptional and post-translational regulation of cellular metabolism, this study has revealed a new link between cellular metabolic sensors and histone modification. Our findings support the existence of a relatively stable protein complex containing OGT, USP7 and MLL5 in the cell nucleus, suggesting physical and functional interplay among O-GlcNAc transferase, deubiquitinase and histone methyltransferase under normal and pathological conditions.

## Supporting Information

S1 Fig Knockdown of OGT led to inhibition of cell proliferation and induction of apoptosis. (A) Proliferation curves of control and OGT knockdown HeLa cells. (B) Flow cytometric analysis of BrdU incorporation in control and OGT knockdown HeLa cells. Anti-BrdU antibody was conjugated with Fluorescein isothiocyanate (FITC) and 7-aminoactinomycin D (7-AAD) was used to stain genomic DNA. (C) Western blotting analysis of cyclin-dependent kinase inhibitor proteins and Cyclin E in OGT knockdown HeLa cells. Actin was used as a loading control. (D) Annexin-V apoptosis assay in OGT knockdown HeLa cells. Annexin-V was conjugated with Fluorescein isothiocyanate (FITC).
(TIF)

S1 Table List of MLL5 associated proteins identified by mass spectrum. (XLS)

## Abbreviations

CHX: cycloheximide
BrdU: bromodeoxyuridine
H3K4: Histone 3 trimethyl at Lysin 4
DAB: 3, 3’-diaminobenzidine
MLL5: Mixed Lineage Leukemia 5
OGT: O-GlcNac Transferase
USP7: Ubiquitin Specific Protease 7
CDKI: cyclin-dependent kinase inhibitor
