# Supplementary figures and images for: Mixed Lineage Leukemia 5 (MLL5) Protein Stability Is Cooperatively Regulated by O-GlcNac Transferase (OGT) and Ubiquitin Specific Protease 7 (USP7)
Source: PLoS One. 2015 Dec 17;10(12):e0145023. doi: 10.1371/journal.pone.0145023 (PMC4683056; doi:10.1371/journal.pone.0145023)

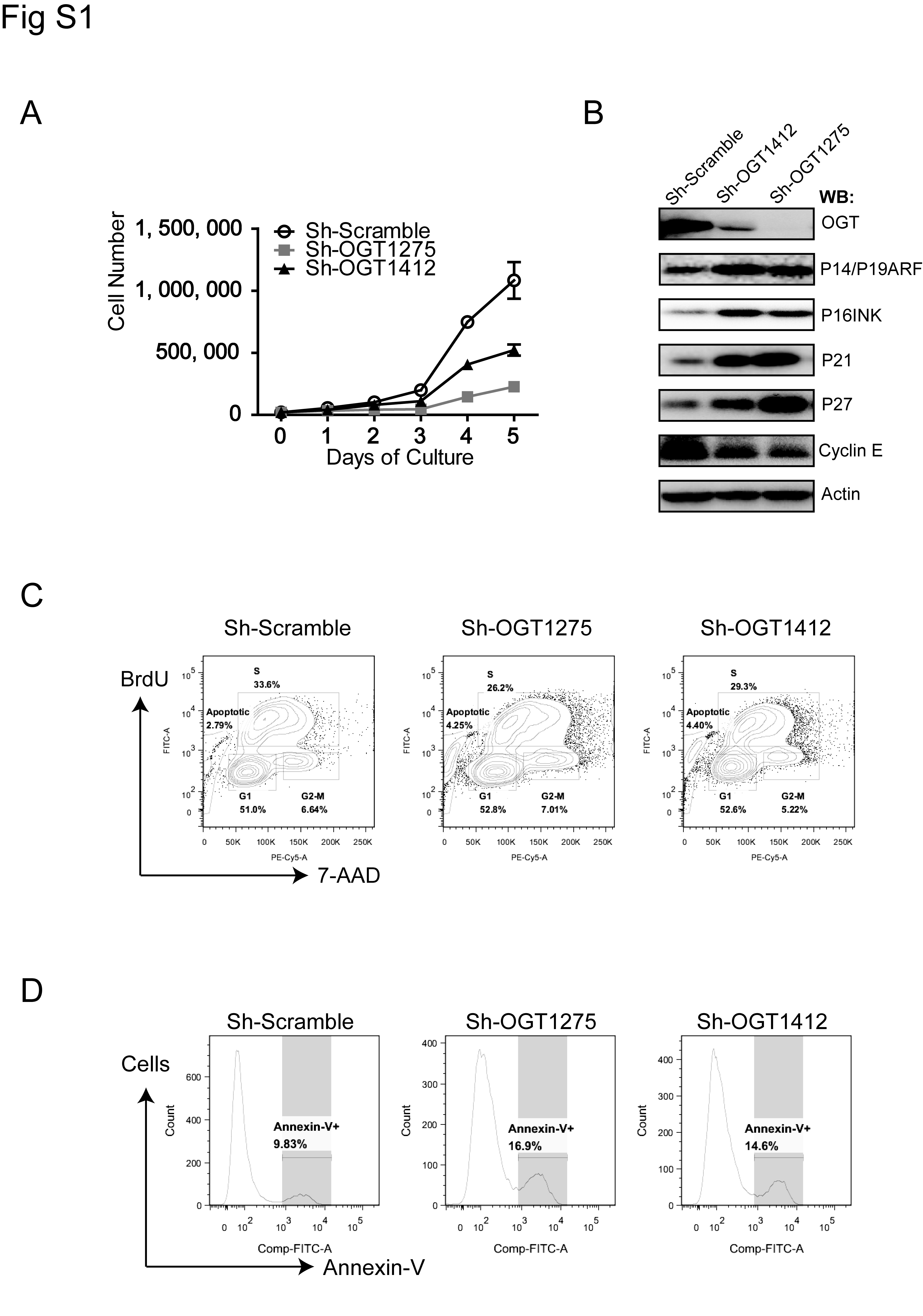

Supplement: S1 Fig — (A) Proliferation curves of control and OGT knockdown HeLa cells. (B) Flow cytometric analysis of BrdU incorporation in control and OGT knockdown HeLa cells. Anti-BrdU antibody was conjugated with Fluorescein isothiocyanate (FITC) and 7-aminoactinomycin D (7-AAD) was used to stain genomic DNA. (C) Western blotting analysis of cyclin-dependent kinase inhibitor proteins and Cyclin E in OGT knockdown HeLa cells. Actin was used as a loading control. (D) Annexin-V apoptosis assay in OGT knockdown HeLa cells. Annexin-V was conjugated with Fluorescein isothiocyanate (FITC). (TIF) [file pone.0145023.s001.tif]
